# Early cardiovascular prevention: the crucial role of nurse-led intervention

**DOI:** 10.1186/s12912-023-01511-6

**Published:** 2023-10-02

**Authors:** Anna Vittoria Mattioli, Sabina Gallina

**Affiliations:** 1 Department of Medical and Surgical Sciences for Children and Adults, University of Modena and Reggio Emilia, Via del pozzo, 71, Modena, 41100 Italy; 2 Istituto Nazionale per le Ricerche Cardiovascolari, Bologna, Italy; 3 Department of Neuroscience, Imaging and Clinical Sciences, “G. d’Annunzio” University of Chieti-Pescara, Chieti, Italy

**Keywords:** Prevention, Nurse, Cardiovascular, Women, Stress, Pregnancy

## Abstract

The present comment on Qiu’s work intends to emphasize two points: (1) Cardiovascular prevention must start early due to the progressive nature of atherosclerosis. (2) growing evidence that coaching performed by nurses leads to effective results. Nurses can intercept the young population who must be sensitized and educated about prevention.

Growing evidence underlines the efficacy of nurse-led intervention in cardiovascular prevention and management of patients with cardiovascular disease (CVD). The article “Nurse-led intervention in the management of patients with cardiovascular diseases: A brief literature review” by Qiu X. analyzes the role of nurses in promoting cardiovascular prevention and in educating patients about correct lifestyles.

There are two points that need to be stressed: (1) Cardiovascular prevention must start early due to the progressive nature of atherosclerosis. (2) the growing evidence that coaching performed by nurses leads to effective results.

Cardiovascular prevention is essential to reduce the impact of cardiovascular disease and for this reason it must be pursued throughout the life course starting from the youngest age. [1, 2] Healthy lifestyles promote longevity but must be adopted early. [1, 3]

The Life’s Simple 7 framework, initially introduced in 2010 by the American Heart Association (AHA), is a set of seven lifestyle factors that are essential for maintaining good cardiovascular health. These factors include: healthy diet, physical activity, no smoking, normal value of body mass index; keeping blood pressure, lipid and bold sugar levels under control. [1] In an update to the framework, the AHA included an eighth factor called sleep health, resulting in the Life’s Essential 8 framework. [4] Adequate sleep is now recognized as an important factor for cardiovascular health, as poor sleep quality and duration have been associated with an increased risk of heart disease and other cardiovascular conditions. By assessing and addressing these eight factors, individuals can adopt a comprehensive approach to promote cardiovascular health and reduce the risk of developing cardiovascular diseases. Prevention is easily adopted in adults after an acute event, while it is much more difficult to promote these habits to young people. It is necessary to intercept young people in the various stages of life to inform them about the risks and educate them, if necessary, on healthy lifestyles. A key moment in the life of young women is pregnancy. [4, 5] In this stage of life, adopting a lifestyle suitable for the health of the woman and the fetus is easy and can be the basis for adopting fundamental behaviors for the prevention of chronic diseases. [4–8] Furthermore, a good pre-pregnancy cardiovascular health reduces the risk of complications during pregnancy for both women and the fetus. During pregnancy, counseling can be effective and give long-term results. Undoubtedly the role of the nurse is central in the management of such a delicate phase. [8–11] In a recent survey Jackson and colleagues found that over 50% of women feel they have not received nutritional counseling or are dissatisfied with nutritional counseling during pregnancy. Perception and satisfaction differ across demographics or socioeconomic status. [7] Women are known to suffer from the impact of social determinants on cardiovascular disease more than men. Social determinants on health (SDoH) refers to the social, economic, and environmental factors that influence health outcomes. [12] These factors can have a profound impact on an individual’s risk of developing CVD. [12] Social isolation, loneliness, discrimination, neighborhood socioeconomic status, violence, and environmental attributes, all fall under the umbrella of SDoH and can contribute to the development and progression of CVD. [12]

Social isolation, fewer social roles, and loneliness can lead to increased stress levels, depression, and unhealthy behaviors, which in turn can contribute to the development of CVD. [13] Discrimination and ethnicity can influence access to healthcare services and resources, leading to disparities in CVD outcomes among different population groups.

Neighborhood socioeconomic status plays a crucial role in health outcomes, including CVD. Lower socioeconomic status is associated with limited access to healthy food options, opportunities for physical activity, and quality healthcare, all of which can increase the risk [14, 15].

Violence and environmental attributes, such as exposure to pollution or lack of green spaces, can also have negative effects on cardiovascular health. Violence and high levels of pollution have been linked to increased stress, inflammation, and hypertension, which are risk factors for CVD. [16–19] Economic circumstances, including income and education level, are important SDoH factors that influence cardiovascular health. Lower socioeconomic status can impact access to healthcare, medication adherence, and lifestyle choices, all of which contribute to CVD risk.

Additionally, early childhood development has been identified as a significant SDoH factor in CVD. Adverse childhood experiences, socioeconomic disadvantages during childhood, and limited access to quality education and healthcare can have long-lasting effects on cardiovascular health in adulthood. [19] Understanding and addressing these social determinants is critical to promoting cardiovascular health and reducing health disparities, and the majority of evidence points to nurses having a role in this action. The other challenge is that women have a reduced perception of their cardiovascular risk and coaching is essential to lead them towards effective prevention. This is a prevention objective that assigns a central role to nurses. Several evidences suggest that coaching carried out by nurses is very effective. [7–9, 20–22].

In conclusion, the nurse led intervention has an important role in cardiovascular prevention especially in the most fragile populations and in women. Early prevention is more effective. Public health must promote actions aimed at intercepting young people and fragile populations.

## Data Availability

No data.
